# Changes in adiponectin system after ventricular assist device in pediatric heart failure

**DOI:** 10.1016/j.jhlto.2023.100041

**Published:** 2024-02

**Authors:** Rosetta Ragusa, Arianna Di Molfetta, Alberto Mercatanti, Letizia Pitto, Antonio Amodeo, Maria Giovanna Trivella, Milena Rizzo, Chiara Caselli

**Affiliations:** a Institute of Clinical Physiology, CNR, Pisa, Italy; b Department of Cardiothoracic Surgery, Ospedale Pediatrico Bambino Gesù, Rome, Italy; c Fondazione Toscana Gabriele Monasterio, Pisa Italy

**Keywords:** pediatric patients, heart failure, VAD, adiponectin system, miRNA

## Abstract

**Background:**

Ventricular assist device (VAD) implant represents a therapeutic option for pediatric patients with end-stage heart failure (HF). Heart unloading by VAD can modify several molecular pathways underlying cardiac function in HF. Among them, the potential role of microRNA (miRNAs) in response to VAD implant is emerging. This study was aimed at investigating in HF pediatric patients the effect of VAD-modified miRNAs on the adiponectin (ADPN) system, known to exert cardioprotective actions.

**Methods:**

ADPN was measured in plasma samples obtained from HF children, before and 1 month after VAD implant, and from healthy control children. miRNA profile and molecules belonging to ADPN system were determined in cardiac biopsies collected at the time of VAD implantation (pre-VAD) and at the moment of heart transplant (post-VAD). An in vitro study using HL-1 cell line was performed to verify the regulatory role of the VAD-modified miRNA on the ADPN system.

**Results:**

VAD implant did not affect circulating and cardiac levels of ADPN, but increased the cardiac mRNA expression of ADPN receptors, including AdipoR1, AdipoR2, and T-cad. AdipoR2 and T-cad were inversely related to the VAD-modified miRNA levels. The in vitro study confirmed the regulatory role of miR-1246 and miR-199b-5p on AdipoR2, and of miR-199b-5p on T-cad.

**Conclusions:**

These data suggest that VAD treatment could regulate the expression of the cardioprotective ADPN system by epigenetic mediators, suggesting that miRNAs have a potential role as therapeutic targets to improve cardiac function in HF pediatric patients.

## Background

Pediatric heart failure (HF) is a clinical syndrome resulting from a broad spectrum of genetic, structural, and neurohormonal abnormalities, characterized by ventricular dysfunction and, as a consequence, impaired oxygenation of organs and tissues.^1^, ^2^, ^3^ It was estimated that each year 11,000 to 14,000 children are hospitalized for HF in the United States.^4^ Congenital heart disease (CHD) and dilated cardiomyopathy (DCM) are indicated as the most common etiologies underlying pediatric HF and the main causes of heart transplants in children.^3^, ^4^, ^5^, ^6^ A dedicated trial aimed to verify the best medical treatments for HF in children has not been yet performed and therapies for pediatric HF are adapted from adult HF treatment, even if the underlying HF etiologies are very different between pediatric and adult patients. Moreover, in end-stage HF children not responders to drug treatment, ventricular assist device (VAD) therapy is now considered a part of standard care as a bridge to heart transplant.^7^, ^8^

**Figure 1:**
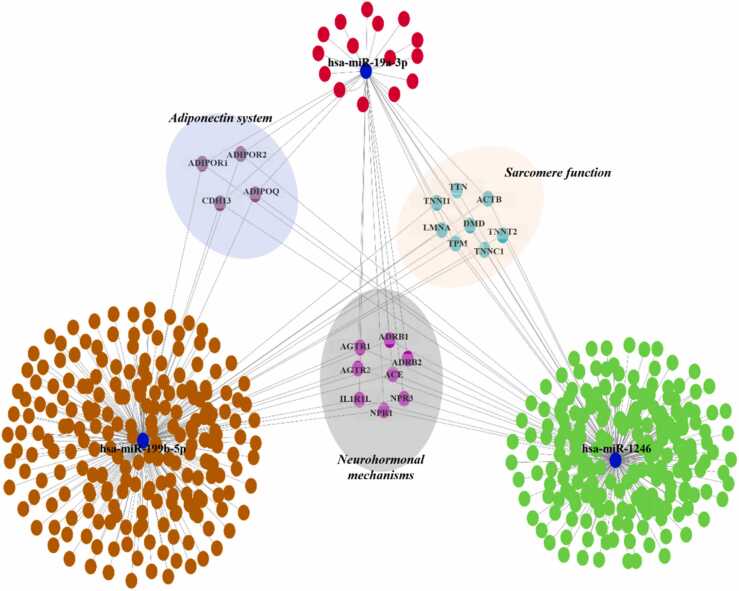
Network analysis of predicted miRNA-targets. The blue circles represent the miRNAs. The red, green, and orange circles represent the potential targets for hsa-miR-19a-3p, hsa-miR-1246, and hsa-miR-199b-5p, respectively. Putative targets potentially regulated through all 3 miRNAs are collected in 3 nodes: adiponectin system; sarcomere function, and neurohormonal mechanisms.

At molecular level, the mechanisms associated with left ventricle (LV) remodeling and pediatric HF progression include the chronic activation of both sympathetic nervous system and renin-angiotensin-aldosterone system (RAAS), the sarcomere proteins’ dysfunction, the cardiac fetal genes re-expression, and the epigenetic modifications.^3^, ^9^ Moreover, VAD treatment could contribute to reverse cardiac remodeling in HF children.^3^ Our previous study suggested that VAD heart unloading was able to modify the expression of specific microRNA (miRNAs) in HF pediatric patients.^9^ The hsa-miR-1246, hsa-miR-199b-5p, and hsa-miR-19a-3p were differentially expressed after VAD implant and were able to regulate the expression of cardiac troponins.^9^ In addition to sarcomere proteins, in silico analysis suggested the adiponectin (ADPN) system, including ADPN and its receptors, as a putative target of selected miRNAs (Figure 1).^9^ ADPN is an adipocyte-derived protein with the anti-inflammatory, anti-hypertrophic, anti-apoptotic, and anti-fibrotic role in the heart.^10^, ^11^, ^12^, ^13^, ^14^, ^15^ The bond of ADPN to its receptors AdipoR1, AdipoR2, and co-receptor T-cadherin (T-cad) mediates these cardioprotective effects.^16^, ^17^, ^18^, ^19^, ^20^, ^21^, ^22^, ^23^, ^24^, ^25^

Thus, this study aimed to examine the ADPN system regulation by VAD-modified miRNAs in pediatric patients with HF. The cardiac expression of the ADPN system was evaluated in HF children at VAD implant and successively at heart transplant. Moreover, the regulatory role of miRNAs in the ADPN system was studied for confirmation by a dedicated in vitro study.

## Materials and methods

### Samples

The study population included 13 HF pediatric patients undergoing VAD implantation as bridge-to-transplantation at the Cardiovascular Department of Ospedale Bambino Gesù of Rome.^9^ This study complied with the principles of the Declaration of Helsinki. Informed consent was given by all parents of children enrolled in this study and the protocol was approved by the Ospedale Bambino Gesù Ethics Committee.

Plasma samples were obtained before the VAD implant and after 1 month of VAD support from 9 HF pediatric patients. Cardiac biopsies were collected at the time of VAD implantation (pre-VAD; N = 8) from the portion of the LV apex and at the time of heart transplant (post-VAD; N = 7) from LV and septum. Plasma samples were collected from a group of 107 healthy children [matched for age and sex] and used as control group. In healthy children, the presence of any significant cardiac disease has been excluded by careful clinical examination and by echocardiography, when necessary, as reported in a previous paper.^26^

### Molecular analysis

Measurement of ADPN levels in plasma samples was performed using Human Adiponectin (ACRP30) ELISA kit (DRG diagnostics, Germany).

Total RNA was extracted from heart biopsies and after cDNA synthesis, the expression of the ADPN system was analyzed by CFX-96 Real-Time PCR detection systems (Bio-Rad). Cardiac miRNA extraction, small cDNA libraries construction, sequencing analysis, identification of miRNA differentially expressed in the heart of children supported by VAD, and the identification of miRNA-gene targets were previously described.^9^

### In vitro study

A transfection study using miRNA mimics was developed to verify the regulatory effect of miRNA on the in silico-predicted mRNA targets. Cardiac Muscle Cell Line (HL-1) (Sigma-Aldrich, St. Louis) was seeded in 6-well plates and transfected with 80 nM of synthetic miRNA mimics. After 48 hours, the expression of the ADPN system was analyzed in HL-1 cells by Real-Time PCR.

### Statistical analysis

Due to not normal distribution of ADPN levels in plasma, the original data were ln-transformed and parametric tests were used. Comparison of ADPN levels between healthy children and pre-VAD group was performed by Student *t*-test, while comparison between pre-VAD and 1-month post-VAD groups was performed by paired Student *t*-test. Differences in cardiac mRNA expression of the ADPN system components between pre- and post-VAD groups were assessed by the Mann-Whitney U test. Correlation among cardiac miRNAs and expression of ADPN system molecules was evaluated by Spearman correlation. Comparison between control and transfected cells was performed by paired Student *t*-test. A *p*-value ≤0.05 was considered significant. All analyses were performed using the SPSS 23 software.

## Results

### Clinical features of HF pediatric patients

Clinical characteristics of the HF pediatric patients at pre- and post-VAD were reported in Table 1. The median age was 29 months and almost half were males. Among them, 70% were affected by DCM, 15% by restrictive cardiomyopathy (RCM), and 15% by left ventricular non-compaction cardiomyopathy (LVNC). An improvement of cardiac function was observed at echocardiography after VAD implantation, while bio-humoral profile was not modified (Table 1).

**Table 1:** Clinical Features of HF Children at the Moment of VAD Implant (Pre-VAD) and the Moment of Heart Transplant (Post-VAD)

	Pre-VAD	Post-VAD	*p*-value
Age, months	29 (5-123)	-	
Male gender, *n*	6 (13)	-	
Weight, kg	9 (4.8-26)	-	
Etiology, *n* (%)			
DCM	9 (70)	-	
LVNC	2 (15)	-	
RCM	2 (15)	-	
LVEF, %	19 (13.75-20.75)	37 (26-45)	0.027
LVEDV, ml	57 (37.5-83)	24 (15.25-30.75)	0.009
LVESV, ml	37 (28.7-71)	14 (8.72-20)	0.020
LVEDD, mm	47 (42.5-56.5)	41 (30.5-49.5)	ns
LVESD, mm	44.5 (39-57)	37 (23-45)	ns
TAPSE, mm	0.985 (0.7-1.25)	0.72 (0.63-1.015)	ns
Creatinine, mg/dl	0.43 (0.275-0.615)	0.37 (0.2-0.5)	ns
Albumin, g/dl	4.2 (3.6-5.25)	4.1 (3.9-4.4)	ns
Glucose, mg/dl	113 (71-128)	91 (87.75-120.75)	ns
C-reactive protein, mg/dl	0.41 (0.11-1.5)	0.8 (0.2-1.7)	ns
Lactate dehydrogenase, U/liter	812 (405-1011)	742 (634-1088)	ns

Abbreviations: DCM, dilated cardiomyopathy; LVEDD, left ventricular end-diastolic diameter; LVEDV, left ventricular end-diastolic volume; LVEF, left ventricular ejection fraction; LVESD, left ventricularend-systolic diameter; LVESV, left ventricular end systolic volume; LVNC, left ventricular non-compaction cardiomyopathy; RCM, restrictive cardiomyopathy; TAPSE, tricuspid annular plane systolic excursion.

### Circulating and cardiac level of ADPN

**Figure 2:**
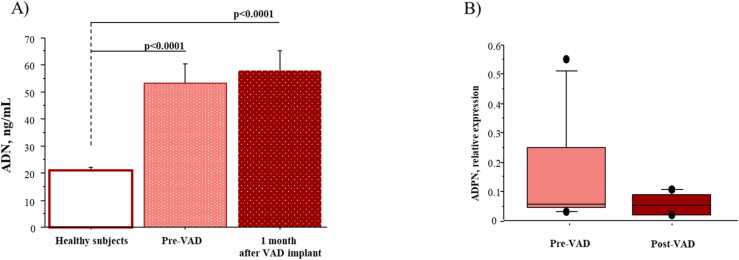
ADPN circulating levels and cardiac gene expression. (A) Comparison of circulating levels of ADPN in the blood collected from healthy children, pediatric patients before VAD implant (pre-VAD), and pediatric patients after 1 month of VAD treatment. (B) Cardiac expression levels of ADPN in children with HF at the moment of VAD implant (pre-VAD) and at removal (post-VAD).

ADPN plasma levels were significantly higher in HF children when compared to healthy subjects and were not modified after 1 month of VAD support (Figure 2A).

The gene expression levels of ADPN in cardiac tissue of pediatric patients with HF were not affected by VAD implantation (Figure 2B).

### ADPN system from in vivo observation to in vitro validation

**Figure 3:**
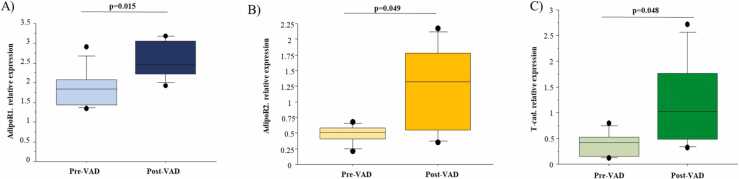
Expression of ADPN receptors at the moment of VAD implantation (pre-VAD) and removal (post-VAD). mRNA expressions of AdipoR1 (A), AdipoR2 (B), and T-cad (C) in cardiac tissue from children patient of pre-VAD group and post-VAD group are shown.

The mRNA levels of the ADPN receptors, AdipoR1, AdipoR2, and T-cad, increased significantly at post-VAD compared to pre-VAD (Figure 3A-C). miRWalk 2.0 database identified ADPN receptors as putative targets of hsa-miR-1246, hsa-miR-199b-5p, and hsa-miR-19a-3p (Figure 1), whose cardiac expression was downregulated at post-VAD compared to pre-VAD.^9^

The relationship among ADPN receptors and miR-1246, hsa-miR-199b-5p, and hsa-miR-19a-3p was evaluated. All cardiac miRNAs were negatively related to AdipoR2, while hsa-miR-1246 was negatively related also to T-cad (Table 2). No relationship was observed among miRNAs and AdipoR1.

**Table 2:** Relation among cardiac miRNAs and ADPN system in HF Children Supported by VAD

	hsa-miR-1246	hsa-miR-19a-3p	hsa-miR-199b-5p
AdipoR1	ns	ns	ns
AdipoR2	Rho = −0.718 *p* = 0.007	Rho = −0.9 *p* = 0.001	Rho = −0.693 *p* = 0.009
T-cad	Rho = −0.51 *p* = 0.049	ns	ns

Abbreviations: HF, heart failure; VAD, ventricular assist device.

**Figure 4:**
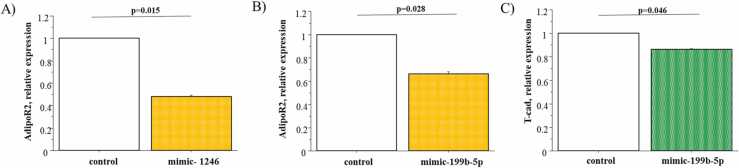
In vitro study. Expression levels of AdipoR2 in HL-1 cell line transfected with mimic-1246 (A), mimic-199b-5p (B), and in control cells (no treated cells). Expression levels of T-cad in HL-1 cell line transfected with mimic-199b-5p and in control cells (C).

To determine whether the expression of ADPN receptors may be regulated by identified cardiac miRNAs, a transfection study was performed treating HL-1 cells with specific miRNA-mimics. Expression levels of AdipoR2 significantly decreased after 48 hours in HL-1 cells transfected with mimic-1246 and mimic-199b-5p compared with untreated cells (Figure 4A, B). T-cad expression decreased only by miRNA-199b-5p mimic use compared to negative control (Figure 4C). AdipoR1 mRNA levels were not modified by transfection using miRNA-mimics.

## Discussion

In this study, for the first time, the effects of previously identified VAD-modified miRNAs^8^ on the molecules belonging to the cardioprotective ADPN system were investigated in HF pediatric patients. These data suggest that VAD implantation could contribute to reverse the cardiac remodeling in HF children, regulating the expression of the cardioprotective ADPN system by epigenetic mediators. Specifically, we found that

– the VAD was able to modify the ADPN system in HF children increasing AdipoR1, AdipoR2, and T-cad mRNA expression;
– hsa-miR-1246, hsa-miR-199b-5p, and hsa-miR-19a-3p affected the cardiac expression of the ADPN system in HF children supported by VAD.

Several evidences in animal and human HF models suggest that the ADPN system is involved in cardiac remodeling by multiple beneficial effects, including anti-hypertrophic, anti-fibrotic, and anti-apoptotic actions.^15^, ^16^, ^25^, ^27^, ^28^ Paradoxically, circulating levels of ADPN increase in HF patients and are related to the worst outcome. This observation is explained by the hypothesis of “ADPN resistance” in which a reduction of ADPN receptors at the tissue level could be responsible for the lack of the ADPN protective effects.^19^, ^20^, ^21^ In cardiomyocytes isolated from C57/BL6 mice, AdipoR1, AdipoR2, and T-cad were significantly reduced after 4 to 8 weeks postmyocardial infarction, suggesting that an altered receptor expression might be responsible for impaired ADPN signaling in late HF stages.^29^ Moreover, AdipoR1 and AdipoR2 protein expression decreased in cardiac biopsies collected from 36 advanced HF adult patients (etiology of HF: *n* = 12 ischemic, *n* = 24 nonischemic) compared with myocardial control samples.^24^ Accordingly, cardiac protein expression of T-cad was downregulated in nonischemic HF adult patients with severe conditions compared to stable HF patients, suggesting for T-cad a role as an indicator of HF severity.^30^ Besides studies in adult patients with HF, this is the first work that investigated the mRNA expression levels of the ADPN system in children with HF. AdipoR1, AdipoR2, and T-cad expression levels at pre-VAD were compared with their values at the moment of heart transplant. Data showed that the mRNA levels of ADPN receptors but not of ADPN were upregulated in the heart of HF children after VAD implantation. The increase of the ADPN receptors in cardiac tissue from HF children after VAD implant could counterbalance the phenomenon of “ADPN resistance” hypothesized for HF patients, leading to a recovery of the ADPN cardioprotective effects. In particular, the ADPN system might exert its cardioprotective effects after 1-month post-VAD, when circulating levels of ADPN returned to pre-VAD levels after an early decline due to the surgery.

Among the molecular mechanism underlying HF progression, epigenetic regulation mediated by miRNAs is emerging.^31^, ^32^ As to the impact of hsa-miRs on the ADPN system in HF, it was observed that hsa-miR-150, selected by bioinformatics approach, was 1.7-fold higher in end-stage HF adult patients compared to control and that the levels of hsa-miR-150 were negatively related to AdipoR2 transcript levels.^23^ As previously reported, hsa-miR-199b-5p, has-miR-19a-3p, and hsa-miR-1246 were identified as downregulated in the heart of HF children after VAD implant.^9^ These VAD-modified miRNAs, able to interfere with cardiac remodeling by troponin expression regulation, have also a regulatory role in the expression of the ADPN system in cardiac tissue, as demonstrated by the dedicated in vitro study. Additionally, the transcription levels of all miRNAs were inversely correlated with ADPN receptors.

This study has some limitations. A comparison between miRNAs and relative target expression of HF pediatric patients with healthy children and with HF adult patients was not performed due to the lack of cardiac tissue from these groups.

Understanding the multitude of altered intracellular networks that are involved in the transition from functional heart to overt HF and demonstrating the effects of VAD and, consequently, the effects of VAD-modified miRNAs on these adverse mechanisms could be of great importance: in fact new therapeutic strategies to reduce HF progression in pediatric patients could be hypothesized from these results. miRNA studies represent a cutting-edge area in molecular biology and medical research with potential translational impact, since deciphering the molecular mechanisms underlying the clinical pathology of disease is the key for implementing personalized medicine. Specifically, as in this study, the identification of specific miRNAs associated with certain diseases provides a basis for developing miRNA-targeted drugs. These drugs might be designed to either inhibit or mimic the action of specific miRNAs, depending on the therapeutic goal. Studies have shown that in cancer, miRNA-based therapy, either by inhibiting an oncomiR or by inducing a tumor suppressor, is an effective treatment. Moreover, the idea of combining miRNA-based therapies with existing treatments is promising. However, a thorough research is required to fully understand the roles of different miRNAs in specific cellular processes and diseases. In addition, the translation of research findings into patient care is a complex and multifaceted process that requires collaboration across disciplines to improving clinical outcomes. Thus, further studies are needed to confirm the regulatory role of VAD-modified miRNAs in ADPN system and to establish the real use of hsa-miR-199b-5p, hsa-miR-19a-3p, and hsa-miR-1246 as therapeutic targets for the treatment of children with HF.

## CRediT authorship contribution statement

RR, MGT, CC: contributed to conceptualization, methodology, investigation and data curation, writing, original draft; LP, MR, AM: contributed to methodology, investigation and data curation; ADM, AA: recruited pediatric patients and contributed to samples collection; ADM, AA, LP, MR, AM, MGT, CC: aided in interpreting the results and reviewed the manuscript. RR, ADM, AM, LP, AA, MGT, MR, CC: final approval of submission.

## Disclosure statement

The authors declare that they have no known competing financial interests or personal relationships that could have appeared to influence the work reported in this paper.

This study was supported partially by grants from the projects SensorART-A Remote Controlled Sensorized ARTificial Heart Enabling Patients Empowerment and New Therapy Approaches (FP7-ICT-2009 Project, Grant Agreement 24863).

## References

[1] Hsu D.T., Pearson G.D. (2009). Heart failure in children: part I: history, etiology, and pathophysiology. Circ Heart Fail.

[2] Hsu D.T., Pearson G.D. (2009). Heart failure in children: part II: diagnosis, treatment, and future directions. Circ Heart Fail.

[3] Ragusa R., Di Molfetta A., Amodeo A., Trivella M.G., Caselli C. (2020). Pathophysiology and molecular signaling in pediatric heart failure and VAD therapy. Clin Chim Acta.

[4] Rossano J.W., Kim J.J., Decker J.A. (2012). Prevalence, morbidity, and mortality of heart failure-related hospitalizations in children in the United States: a population-based study. J Card Fail.

[5] Kirk R., Dipchand A.I., Rosenthal D.N. (2014). The International Society for Heart and Lung Transplantation Guidelines for the management of pediatric heart failure: executive summary. J Heart Lung Transplant.

[6] Adorisio R., Pontrelli G., Cantarutti N. (2022). Heart rate reduction as a marker to optimize carvedilol treatment and enhance myocardial recovery in pediatric dilated cardiomyopathy. Front Physiol.

[7] Zafar F., Castleberry C., Khan M.S. (2015). Pediatric heart transplant waiting list mortality in the era of ventricular assist devices. J Heart Lung Transplant.

[8] Napoli C., Grimaldi V., De Pascale M.R., Sommese L., Infante T., Soricelli A. (2016). Novel epigenetic-based therapies useful in cardiovascular medicine. World J Cardiol.

[9] Ragusa R., Di Molfetta A., Del Turco S. (2021). Epigenetic regulation of cardiac troponin genes in pediatric patients with heart failure supported by ventricular assist device. Biomedicines.

[10] Ding M., Rzucidlo E.M., Davey J.C. (2012). Adiponectin in the heart and vascular system. Vitam Horm.

[11] Baltrūnienė V., Rinkūnaitė I., Bogomolovas J. (2020). The role of cardiac T-cadherin in the indicating heart failure severity of patients with non-ischemic dilated cardiomyopathy. Medicina (Kaunas).

[12] Shibata R., Sato K., Pimentel D.R. (2005). Adiponectin protects against myocardial ischemia-reperfusion injury through ampk- and cox-2-dependent mechanisms. Nat Med.

[13] Mitsuhashi H., Yatsuya H., Tamakoshi K. (2007). Adiponectin level and left ventricular hypertrophy in Japanese men. Hypertension.

[14] Okamoto H. (2009). Can adiponectin be a novel metabolic biomarker for heart failure?. Circ J.

[15] Caselli C., D'Amico A., Cabiati M., Prescimone T., Del Ry S., Giannessi D. (2014). Back to the heart: the protective role of adiponectin. Pharmacol Res.

[16] Caselli C., Lionetti V., Cabiati M. (2012). Regional evidence of modulation of cardiac adiponectin level in dilated cardiomyopathy: pilot study in a porcine animal model. Cardiovasc Diabetol.

[17] Denzel M.S., Scimia M.C., Zumstein P.M., Walsh K., Ruiz-Lozano P., Ranscht B. (2010). T-cadherin is critical for adiponectin-mediated cardioprotection in mice. J Clin Invest.

[18] Yamauchi T., Kamon J., Ito Y. (2003). Cloning of adiponectin receptors that mediate antidiabetic metabolic effects. Nature.

[19] Kistorp C., Faber J., Galatius S. (2005). Plasma adiponectin, body mass index, and mortality in patients with chronic heart failure. Circulation.

[20] Shibata R., Ouchi N., Murohara T. (2009). Adiponectin and cardiovascular disease. Circ J.

[21] Mattu H.S., Randeva H.S. (2013). Role of adipokines in cardiovascular disease. J Endocrinol.

[22] Kamareddine L., Ghantous C.M., Allouch S. (2021). Between inflammation and autophagy: the role of leptin-adiponectin axis in cardiac remodeling. J Inflamm Res.

[23] Kreth S., Ledderose C., Schütz S. (2014). MicroRNA-150 inhibits expression of adiponectin receptor 2 and is a potential therapeutic target in patients with chronic heart failure. J Heart Lung Transplant.

[24] Khan R.S., Kato T.S., Chokshi A. (2012). Adipose tissue inflammation and adiponectin resistance in patients with advanced heart failure: correction after ventricular assist device implantation. Circ Heart Fail.

[25] Caselli C., Cantinotti M., Del Ry S. (2012). Adiponectin plasma levels decrease after surgery in pediatric patients with congenital heart disease. Clin Biochem.

[26] Ragusa R., Prontera C., Di Molfetta A. (2018). Time-course of circulating cardiac and inflammatory biomarkers after ventricular assist device implantation: comparison between paediatric and adult patients. Clin Chim Acta.

[27] Zhang N., Wei W.Y., Liao H.H. (2018). AdipoRon, an adiponectin receptor agonist, attenuates cardiac remodeling induced by pressure overload. J Mol Med (Berl).

[28] Amin R.H., Mathews S.T., Alli A., Leff T. (2010). Endogenously produced adiponectin protects cardiomyocytes from hypertrophy by a PPARgamma-dependent autocrine mechanism. Am J Physiol Heart Circ Physiol.

[29] Wang Y., Gao E., Lau W.B. (2015). G-protein-coupled receptor kinase 2-mediated desensitization of adiponectin receptor 1 in failing heart. Circulation.

[30] Baltrūnienė V., Rinkūnaitė I., Bogomolovas J. (2020). The role of cardiac T-cadherin in the indicating heart failure severity of patients with non-ischemic dilated cardiomyopathy. Medicina (Kaunas).

[31] Matkovich S.J., Van Booven D.J., Youker K.A. (2009). Reciprocal regulation of myocardial microRNAs and messenger RNA in human cardiomyopathy and reversal of the microRNA signature by biomechanical support. Circulation.

[32] Leptidis S., El Azzouzi H., Lok S.I. (2013). A deep sequencing approach to uncover the miRNOME in the human heart. PLoS One.

